# Anticoagulation Resumption After Intracerebral Hemorrhage

**DOI:** 10.1007/s11883-018-0733-y

**Published:** 2018-05-21

**Authors:** Yan-guang Li, Gregory Y. H. Lip

**Affiliations:** 1 Institute of Cardiovascular Sciences, University of Birmingham, Birmingham, England UK; 2 Department of Cardiology, Chinese PLA Medical School, Beijing, China; 3 Aalborg Thrombosis Research Unit, Department of Clinical Medicine, Aalborg University, Aalborg, Denmark

**Keywords:** Intracerebral hemorrhage, Anticoagulation, Resumption, Warfarin, Non-vitamin K antagonist oral anticoagulants

## Abstract

**Purpose of review:**

Decision-making on resuming oral anticoagulant (OAC) after intracerebral hemorrhage (ICH) evokes significant debate among clinicians. Such patients have been excluded from randomized clinical trials. This review article provides a comprehensive summary of the evidence on anticoagulation resumption after ICH.

**Recent findings:**

OAC resumption does not increase the risk of recurrent ICH and can also reduce the risk of all-cause mortality. OAC cessation exposes patients to a significantly higher risk of thromboembolism, which could be reduced by resumption. The optimal timing of anticoagulation resumption after ICH is still unknown. Both early (< 2 weeks) and late (> 4 weeks) resumption should be reached only after very careful assessment of risks for ICH recurrence and thromboembolism. The introduction of new oral anticoagulants and other interventions, such as left atrial appendage closure, has provided some patients with more alternatives.

**Summary:**

Given the lack of high-quality evidence to guide clinical decision-making, clinicians must carefully balance the risks of thromboembolism and recurrent ICH in individual patients. We propose a management approach which would facilitate the decision-making process on whether anticoagulation is appropriate, as well as when and how to restart anticoagulation after ICH.

## Introduction

Intracerebral hemorrhage (ICH) is associated with a high risk of mortality and stroke, as well as recurrent ICH [1•]. Of note, ICH is also the most devastating adverse event in patients receiving oral anticoagulants (OAC) [2]. Among patients on anticoagulation, the annual incidence of ICH varies between 0.6 and 1.0% [3]. Indeed, anticoagulation-related ICH is more severe and associated with more extensive hemorrhage and higher mortality rate, compared with spontaneous ICH [4].

Anticoagulation is supported by Class I guidelines for patients with atrial fibrillation (AF) and high risk of ischemic stroke (IS) and systemic embolism (SE), mechanical prosthetic valves, or those at high risk of deep venous thrombosis (DVT) and pulmonary embolism (PE). Decision-making on resuming anticoagulation after ICH evokes significant debate among clinicians. Such patients have been excluded from randomized clinical trials of stroke prevention in AF. As with decision-making with anticoagulation therapy in any patient, carefully balancing the risks of thromboembolism and bleeding, especially the risk of recurrent ICH, is the primary concern when making this decision in patients who have experienced a recent ICH [5]. The lack of high-quality evidence, however, makes the decision-making challenging for clinicians and quite variable in practice [6].

**Fig. 1:**
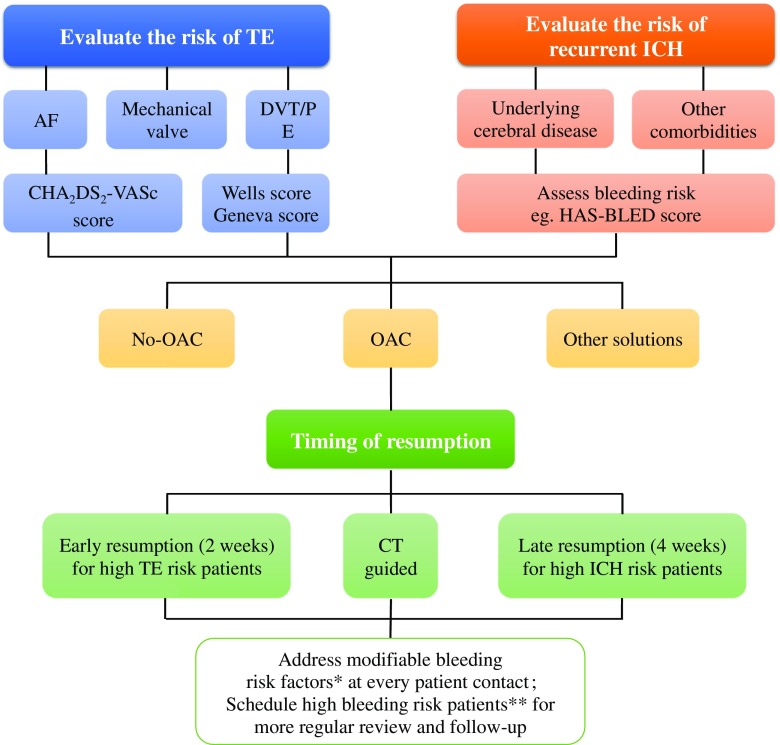
Flowchart of decision-making regarding OAC resumption in patients with recent ICH. AF = atrial fibrillation; CT = computed tomography; CHA_2_DS_2_-VASc = congestive heart failure, hypertension, age ≥ 75 years, type 2 diabetes, previous stroke/transient ischemic attack/thromboembolism, vascular disease, age 65~74 years, and gender category; DVT = deep venous thrombosis; HAS-BLED = hypertension, abnormal renal/liver function, stroke, bleeding history or predisposition, labile international normalized ratio (INR), elderly, drugs/alcohol concomitantly; ICH, intracerebral hemorrhage; OAC, oral anticoagulant; PE = pulmonary embolism; TE = thromboembolism. A single asterisk indicates modifiable bleeding risk factors that include uncontrolled blood pressure, labile INRs (if on warfarin), concomitant aspirin/NSAID use, alcohol excess. Double asterisks indicate high risk patients can be defined as HAS-BLED score ≥ 3

At a simplistic level, we propose three steps to make such a decision (Fig. 1): first, evaluate the individual’s risk of thromboembolism and hemorrhage [7]; second, choose the optimal anticoagulant and appropriate timing to reinitiate anticoagulation [8]; and third, reduce the risk of recurrent hemorrhage through controlling modifiable risk factors, such as uncontrolled hypertension, anemia, renal dysfunction, diabetes, and heart failure (HF) [9, 10].

Nevertheless, this approach towards decision-making could also be difficult in clinical practice, because of the lack of risk evaluation tools for complicated clinical conditions, the insufficient evidence from the randomized clinical trials, and the existence of shared risk factors for both thromboembolism and hemorrhage.

This review article aims to provide an up-to-date overview regarding the pros and cons of restarting anticoagulation after ICH. We also propose a management approach which would facilitate the decision-making process on whether anticoagulation is appropriate, as well as when and how to restart anticoagulation after ICH.

## The Risks of Recurrent ICH, Thromboembolism, and Mortality

Current guidelines recommended that the decision on restarting OAC should be taken by a multidisciplinary team of stroke physicians, neurologists, cardiologists, neuroradiologists, and neurosurgeons [11]. This decision mainly depends on an individual’s risk of recurrent ICH versus risk of thromboembolism. The relative risk of mortality is also a major concern.

### Risk of Recurrent ICH After OAC Resumption

In non-anticoagulated patients with a history of ICH, the 1-year risk of recurrent ICH ranges from 0 to 8.6% [12]. In patients resuming OAC, this number ranges from 2.5 to 8% [12]. For example, in one multicenter study (*n* = 267), warfarin resumption was associated with an annual ICH recurrence rate of 2.56% [13]. Whether OAC resumption per se truly increases the risk of recurrent ICH is still debatable, given the many associated comorbidities related to ICH recurrence.

The majority of studies demonstrate that OAC resumption did not increase the risk of recurrent ICH [14, 15••, 16–19] (see Table 1). In one retrospective cohort study (*n* = 160), recurrent ICH occurred with higher frequency after OAC resumption, but this was statistically nonsignificant compared with patients who did not resume OAC (7.6 vs. 3.7%, *p* = 0.48) [14]. Also, OAC resumption did not increase the risk of ICH in a Danish nationwide cohort study [15••]. In another large observational cohort (*n* = 2415), warfarin resumption after incident ICH was associated with similar risk of recurrent ICH to non-resumption of warfarin [17••]. These results were confirmed by a recent systematic review and meta-analysis, including eight studies and 5306 ICH patients, in which reinstatement of OAC had a similar risk of recurrent ICH to non-OAC restarters [18].

**Table 1:** Studies evaluating risk of hemorrhage and thromboembolism after ICH

First Author	Design	No.	Patients with OAC resumption				Patients without OAC resumption			HR (95% CI) of OAC resumption		
			Time of OAC restarting (days)	Incidence of recurrent ICH^*^	Incidence of TE^*^	Incidence all-cause mortality^*^	Incidence of recurrent ICH^*^	Incidence of TE^*^	Incidence all-cause mortality^*^	ICH	TE	All-cause mortality
Ottosen [1•]	Population-based cohort	6369	N/A	N/A	N/A	N/A	N/A	N/A	N/A	0.90 (0.44–1.82)	0.58 (0.35–0.97)	0.59 (0.43–0.82)
Witt [14]	Retrospective cohort	160	14	7.6	3.7	18.5	3.7	12.3	31.1	0.47 (0.10–2.30)	0.28 (0.06–1.27)	0.76 (0.30–1.89)
Nielsen [15••]	Nationwide cohort	1752	34	8.0	5.3	9.7	8.6	10.4	19.1	0.91 (0.56–1.49)	0.59 (0.33–1.03)	0.55 (0.37–0.82)
Kuramatsu [16]	Nationwide cohort	719	31	3.9	5.2	8.2	3.9	15.0	37.5	N/A	N/A	0.26 (0.13–0.53)
Nielsen [17••]	Nationwide cohort	2415	31	5.8	3.3	19.6	5.3	8. 9	35.5	1.31 (0.68–2.50)	0.43 (0.21–0.86)	0.51 (0.37–0.71)
Santosh [18]	Meta-analysis	5306	N/A	N/A	N/A	N/A	7.8	N/A	N/A	1.01 (0.58–1.77)	0.34 (0.25–0.45)	N/A
Poli [19]	Nationwide cohort	244	N/A	N/A	2.0	3.0	N/A	6.0	8.0	N/A	0.19 (0.06–0.60)	0.17 (0.06–0.45)
Park [20]	Retrospective	528	117	1.4	2.4	1.4	0	8.3	4.8	N/A	0.19 (0.08–0.47)	N/A

*HR* hazard ratio, *CI* confidence interval, *ICH* intracerebral hemorrhage, *OAC* oral anticoagulants, *TE* thromboembolism

^*^Per 100 person-years

Nevertheless, some contradictory results exist. For example, a retrospective study (*n* = 428) showed that OAC restarting increased the risk of major bleeding (5.5 vs. 3.1 per 100 patient-years, *p* = 0.024), and recurrent ICH was observed only in patients with OAC use [20]. Different study designs and selection biases may explain the contradictory results. In many studies, only the patients with “less severe” ICH, that is ICH with smaller volume of hemorrhage and mild functional changes, could have received OAC resumption, hence leading to a lower recurrent ICH risk [21, 22].

For clinical practice, different patient profiles may lead to a varying risk of ICH recurrence, and therefore, individualized evaluation is critical [23]. We suggest that risk factors for recurrent ICH should be considered before deciding OAC resumption [24, 25]. Currently, there are some well-identified risk factors for recurrent ICH (Table 2). For example, the location of ICH is a significant risk factor. Lobar hemorrhage has a higher ICH recurrent rate compared with hemorrhage in a deep cortical location (22 vs. 4% for cumulative 2-year rate, *p* = 0.007) [26].

**Table 2:** Clinical risk factors of recurrent ICH and thromboembolism

Risk factor category	Risk factors	Modifiable risk factors
Risk factors for recurrent ICH	Large area ICH, ICH history, lobar ICH location, cerebral microbleeds, amyloid angiopathy, arteriovenous malformation, cerebral aneurysm, lacunar infarcts, leukoaraiosis, Asian population	Alcohol, tobacco, anemia, hepatic disease, high risk of fall
Risk factors for both ICH and thromboembolism	Elderly, coagulopathy, previous IS, malignancy	Hypertension, diabetes, kidney dysfunction, labile INR
Risk factors of thromboembolism	AF, HF, vascular disease, mechanical heart valve, VTE history, female sex, recent surgery	Decreased ambulation

*AF* atrial fibrillation, *HF* heart failure, *INR* international normalized ratio, *IS* ischemic stroke, *VTE* venous thromboembolism; other abbreviations see Table 1

In postoperative (neurosurgery for ICH) patients with spontaneous ICH, diabetes mellitus (odds ratio [OR], 2.72; 95% CI, 1.01–7.35) has been related to recurrent ICH [27]. In other studies, patients with hepatic C virus infection had increased risk of ICH (HR, 1.60; 95% CI, 1.24–2.06) [28], as has severe hypertension (systolic blood pressure ≥ 160 mmHg or diastolic blood pressure ≥ 110 mmHg), which was associated with a sixfold increased risk of ICH [29]. In addition, patients with recent intracranial microbleeds had substantial risk of incident ICH [30]. Leukoaraiosis was also related with high risk of significant ICH (relative risk [RR], 1.65; 95% CI, 1.26–2.16) [31]. For these two risk factors, brain imaging evidence provided by computed tomography (CT) or magnetic resonance imaging (MRI) is required.

Ethnicity is also a major risk factor for ICH. Asian populations had higher risk of ICH in the major trials of the non-VKA oral anticoagulants (NOACs) [32–35]. For example, in the RE-LY (Randomized Evaluation of Long-term Anticoagulant Therapy) trial, Asian populations had higher risk of ICH in both of the warfarin (1.10 vs. 0.71%/year) and dabigatran (0.45 vs. 0.29%/year) arms compared with non-Asian populations [35]. Further, the East Asian population had over twofold risk of ICH compared with non-East Asian ones in the ARISTOTLE (Apixaban for Reduction in Stroke and Other Thromboembolic Events in Atrial Fibrillation) trial (warfarin, 1.88 vs. 0.67%/year; apixaban, 0.67 vs. 0.30%/year) [33].

Modifiable bleeding risk factors should be addressed at every opportunity: for example, uncontrolled blood pressure and concomitant use of aspirin or non-steroidal anti-inflammatory drugs. An approach based on modifiable bleeding risk factors alone, however, is an inferior strategy to using a formal bleeding risk score to assess bleeding risk [9, 10, 36].

Several bleeding risk scores have been proposed (Tables 3 and 4), including the mOBRI score (modified Outpatient Bleeding Risk Index) [37], the HEMORR_2_HAGES score (Hepatic or Renal Disease, Ethanol Abuse, Malignancy, Older Age, Reduced Platelet Count or Function, Re-Bleeding Risk, Hypertension, Anemia, Genetic Factors, Excessive Fall Risk, Stroke) [38], the Shireman score [39], the HAS-BLED score (Hypertension, Abnormal Renal/Liver Function, Stroke, Bleeding History or Predisposition, Labile INR, Elderly, Drugs/Alcohol Concomitantly) [40], the ATRIA score (Anticoagulation and Risk Factors In Atrial Fibrillation) [45], and the ORBIT score (national Outcomes Registry for Better Informed Treatment of Atrial Fibrillation) [46]. Of the various bleeding risk scores, the HAS-BLED score has been validated to predict ICH and recurrent ICH after first spontaneous ICH [10, 47, 48]. For example, in patients with spontaneous ICH, the risk of ICH recurrence increased with the HAS-BLED score, ranging from 1.37% per patient-year for a score of 1 to 2.90% per patient-year for a score of 4 [48].

**Table 3:** Risk scores for evaluating anticoagulated individual’s risk of bleeding

Risk score	C statistic (95% CI)	Variables	Risk category
mOBRI [35]	0.78 (N/A)	Age ≥ 65, previous stroke, gastrointestinal bleed, ≥ 1 of the following comorbidities (recent MI, hematocrit < 30%, creatinine > 1.5 mg/ml, diabetes)	Low risk: 0; intermediate risk: 1–2; high risk: ≥ 3
HEMORR_2_HAGE [36]	0.67 (N/A)	Prior bleed (2 points), hepatic or renal disease, ethanol abuse, malignancy, age ≥ 75, reduced platelet count or function, uncontrolled hypertension, anemia, genetic factor, excessive fall risk, stroke	Low risk: 0–1; intermediate risk: 2–3; high risk: ≥ 4
Shireman [37]	0.63 (N/A)	Age ≥ 70 (0.49 points), female (0.32 points), remote bleed (0.58 points), recent bleed (0.62 points), alcohol/drug abuse (0.71 points), diabetes (0.27 points), anemia (0.86 points), antiplatelet (0.32 points)	Low risk: 0–1.07; intermediate risk: 1.07–2.19; high risk: ≥ 2.19
HAS-BLED [38]	0.72 (0.64–0.79)	Uncontrolled systolic blood pressure, abnormal renal/liver function, stroke, bleeding history, labile international normalized ratio, age ≥ 65, drug, concomitant alcohol	Low risk: 0–1; intermediate risk: 2; high risk: ≥ 3
ATRIA [39]	0.74 (0.70–0.78)	Anemia (3 points), severe renal disease (eGFR < 30 ml/min or dialysis-dependent) (3 points), age ≥ 75 (2 points), previous bleed, hypertension	Low risk: 0–3; intermediate risk: 4; high risk: ≥5
ORBIT [40]	0.69 (0.63–0.74)	Age ≥ 74, insufficient kidney function (eGFR < 60 ml/min), antiplatelet, bleeding history (2 points), anemia (2 points), abnormal hemoglobin (< 13 mg/dL for males and < 12 mg/dL for females) (2 points)	Low risk: 0–2; intermediate risk: 3; high risk: ≥ 4

*ATRIA* anticoagulation and risk factors in atrial fibrillation, *CI* confidence interval, *eGFR* estimated glomerular filtration rate, *MI* myocardial infarction, *mOBRI* modified outpatient bleeding risk index, *ORBIT* outcomes registry for better informed treatment of atrial fibrillation

**Table 4:** Risk scores for evaluating individuals risk of thromboembolism

Risk score	Application	C statistic (95% CI)	Variables	Risk category
CHADS_2_ [41]	AF patients	0.82 (0.80–0.84)	Congestive heart failure, hypertension, age ≥ 65, diabetes, IS/TIA/SE (2 points)	Low risk: 0–1; intermediate risk: 2–3; high risk: ≥ 4
CHA_2_DS_2_-VASc [42]	AF patients	0.61 (0.51–0.70)	Congestive HF, hypertension, age ≥ 75 (2 points), diabetes, IS/TIA/SE (2 points), vascular disease, age 65–74, female gender	Low risk: 0; intermediate risk: 1; high risk: ≥ 2
Modified Wells score [43]	DVT/PE	N/A	Active cancer, immobilization, recent bedridden, tenderness along the deep venous system, entire leg swollen, calf swelling, pitting edema, collateral superficial veins	Low risk: 0; intermediate risk: 1–2; high risk: ≥ 3
Revised Geneva score [44]	DVT/PE	0.79 (0.76–0.81)	Age 60–79, age ≥ 80 (2 points), previous PE/DVT (2 points), recent surgery (3 points), pulse rate > 100/min, PaCO_2_ < 4.8 kPa (2 points), PaCO_2_: 4.8–5.19 kPa, PaO_2_ < 6.5 kPa (4 points), PaO_2:_ 6.5–7.99 kPa (3 points), PaO_2:_ 8.9–9.49 kPa (2 points), PaO_2:_ 9.5–10.99 kPa, pleatlike atelectasis, elevation of a hemidiaphragm on chest X-ray film	Low risk: 0–4; intermediate risk: 5–8; high risk: ≥ 9

*CHADS*_*2*_ congestive heart failure, hypertension, age ≥ 65 years, type 2 diabetes, previous stroke/transient ischemic attack/thromboembolism, *CHA*_*2*_*DS*_*2*_*-VASc* congestive heart failure, hypertension, age ≥ 75 years, type 2 diabetes, previous stroke/transient ischemic attack/thromboembolism, vascular disease, age 65~74 years, and gender category; *DVT* = deep venous thromboembolism, *IS* ischemic stroke, *PE* pulmonary embolism, *SE* systemic embolism, *TIA* transient ischemic attack; other abbreviations see Table 1

Although some patients have significant risk factors or a high bleeding risk score, these need not be considered absolute contraindications. Indeed, a high bleeding risk score should be used to identify the patients at risk for more careful review and early follow-up after OAC resumption, and not used as an excuse to withhold OAC [49], because during OAC cessation the patients face a higher risk of thromboembolism, which can increase mortality [50].

### Risk of Thromboembolism During OAC Cessation

OAC resumption is important for the patients at high risk of thromboembolism, such as those with prosthetic mechanical valve, high risk of PE, and AF patients with a high CHA_2_DS_2_-VASc score (congestive HF, hypertension, age ≥ 75 years, type 2 diabetes, previous stroke/TIA/thromboembolism, vascular disease, age 65~74 years, and gender category), i.e., CHA_2_DS_2_-VASc score ≥ 4. In a recent systematic review and meta-analysis of restarting OAC after ICH, AF is the most common reason for anticoagulation (34.7–77.8%), followed by prosthetic heart valve (2.6–27.8%), venous thromboembolism (7.9–20.8%), and previous IS (3.7–71.8%) [18]. In other studies, the most common reason for OAC resumption after ICH was mechanical heart valve (39–68%) [14, 16]. Undoubtedly, the prolonged cessation of OAC after ICH would expose these high-risk patients to a greater risk of thromboembolism.

For example, the risk of major thromboembolism among patients (*n* = 13,000) with prosthetic heart valves and not on OAC was 4.0 per 100 patient-years (95% CI, 2.9–5.2) [51]. Mitral valve prosthesis is associated with a fivefold higher incidence of valve thrombosis and 1.5 times greater incidence of thromboembolism [52]. The combination of double mechanical prosthesis was related to an even higher risk of thromboembolism (91%) [12]. Also, the risk of DVT was 2 to 15% in patients with recent ICH [53]. PE occurs in 1 to 5% of recent ICH patients in which anticoagulation was stopped [12]. Given the high risk of recurrent venous thromboembolism, these patients may require antithrombotic therapy despite the risk of recurrent ICH.

OAC resumption can reduce the risk of thromboembolism. For example, a retrospective study demonstrated that OAC in AF patients after ICH was associated with a significantly reduced incidence of thromboembolism (RR, 0.19; 95% CI, 0.11–0.54) [20]. In a nationwide cohort (*n* = 2415), warfarin resumption had a lower rate of IS and SE in AF patients with hemorrhagic stroke (HR, 0.49; 95% CI, 0.24–1.02) [17••]. In a Danish cohort (*n* = 1725), the patients with OAC resumption were associated with 41 to 54% lower risk of IS/SE and all-cause mortality, compared with no OAC treatment [15••]. These results were also confirmed in the Danish nationwide registries (*n* = 6369) (HR, 0.58; 95% CI, 0.35–0.97) [1•], another Danish study (HR, 0.55; 95%, 0.39–0.78) [15••] and in a German cohort (5.2 vs. 15% per 100 patient-years, *p* < 0.001) [16]. In a recent meta-analysis about OAC resumption after ICH, reinitiation of OAC resulted in a significantly lower risk of thromboembolic complications (HR, 0.34; 95% CI, 0.25–0.45) [18]. Also, the MUCH-Italy study (Multicentre Study on Cerebral Hemorrhage in Italy) showed an 81% reduced risk of thromboembolism among patients restarted OAC after ICH [19].

Despite the high risk of thromboembolism and the efficiency of anticoagulation, a large proportion of ICH survivors often do not resume OAC [1•, 15••, 54, 55]. Although some prior studies suggest that OAC could be discontinued safely for a certain period without significant high risk of thromboembolism, the duration of this period is less certain [41]. For example, in the REVERSE-AD study (Reversal Effects of Idarucizumab on Active Dabigatran), the majority of the thrombotic events occurred in patients who had not resumed OAC in the first 30 days after ICH [42]. Thus, although immediate OAC resumption is not appropriate for most patients, the duration of withholding OAC should be carefully considered and balanced against the risk of recurrent thromboembolism.

There are some well-identified risk factors for thromboembolism (see Table 2), and several risk scoring systems have been proposed to evaluate the individual’s risk of thromboembolism, such as the CHADS_2_ [43] and CHA_2_DS_2_-VASc scores in AF [44], and the modified Wells score [56] and the revised Geneva score in venous thromboembolism [57] (Table 4). Identifying these risk factors and risk score would be useful to predict the individuals’ risk of thromboembolism.

### OAC Resumption and Risk of Mortality

The 1-year risk of mortality in patients with ICH who restarted OAC ranges from 2.5 to 48% (Table 1). Previous studies demonstrated that OAC resumption after ICH had significantly lower risk of death [1•, 16]. In a large Danish observational study, warfarin resumption reduced the rate of mortality in patients with hemorrhagic stroke (Table 1) [17••]. More recently, the MUCH study showed a reduced mortality rate in patients with warfarin resumption compared with those not on OAC [19]. While OAC resumption in patients with lobar ICH is associated with higher risk of recurrent ICH, it is also associated with decreased mortality (HR, 0.29; 95% CI, 0.17–0.45) [58].

## Timing of Restarting OAC

In patients who require OAC after the acute period of ICH, it is important to choose the right timing for restarting OAC. The opinion about the timing of reinitiating OAC (warfarin) ranges from 3 days to 30 weeks [59]. In a Japanese survey, for example, 28% patients restarted OAC within the first week, 25% during the second week, 28% between 3 or 4 weeks, and 18% after 4 weeks [60].

In the Danish nationwide cohort (*n* = 1725), OAC was restarted at 2–10 weeks after ICH [15••]. Other evidence supports that OAC can be restarted 4–8 weeks after ICH if the cause of ICH has been treated [61]. Also, restarting OAC at 4 weeks after ICH was associated with 42 to 59% lower risk of recurrent ICH [15••]. In a multicenter retrospective study in Germany (*n* = 719), anticoagulation was restarted at median of 31 days, and these patients had fewer ischemic complications (5.2 vs. 15.0%, *p* < 0.001), decreased risk of unfavorable functional outcome (RR, 0.55; 95% CI, 0.39–0.78), and similar hemorrhagic complications (8.1 vs. 6.6%, *p* = 0.48), compared with no OAC resumption [16]. Another study also demonstrated that OAC resumption at 2 weeks after ICH seemed reasonable, resulting in less clinical events including thromboembolism event [20].

Although heparin or OAC may probably be safe to be restarted after day 7 post-ICH, without increasing the risk of ICH [62], any early OAC resumption (<2 weeks) should be cautiously considered. An observational study demonstrated that early OAC resumption (<2 weeks) could not improve the composite outcome (i.e., thromboembolic events, major bleeding events, and all-cause mortality), particularly because of an increased risk of major bleeding events [20]. Given these risks, some researchers have suggested that OAC should be avoided in the first 2 weeks after OAC-associated parenchymal ICH and resumption at 4 weeks if the cause of ICH has been amended or in patients with small ICH and high thromboembolic risk [63].

Hence, the timing of OAC restarting depends on individual clinical condition (i.e., the risks of thromboembolism and likelihood of recurrent ICH). For example, in patients with brainstem or cerebellar ICH, the timing should be delayed at least 8–10 weeks after the event [63]. On the other hand, in patients with prosthetic mechanical valves, who have a (very) high risk of thromboembolism, OAC is suggested to be resumed at 2 weeks after the onset of ICH or sooner if the hemorrhage burden is small and causative mechanism treated or stabilized.

When considering the timing of restarting anticoagulation, a brain CT scan and MRI can help to confirm the resolution of ICH [64]. Restarting OAC without the confirmation of ICH resolution has been related to increased composite outcome (i.e., thromboembolic events, major bleeding events, and all-cause mortality) in a retrospective study (RR, 4.40; 95% CI, 1.02–19.04) [20]. Nevertheless, selection bias such that subjects with less severe ICH often restart OAC earlier than severe ones, may have led to these findings of better outcomes.

### The Choice of Anticoagulant

Currently, we have a number of anticoagulants that are approved for use for atrial fibrillation and PE/DVT. The vitamin K antagonists (VKAs) are widely used in patients with AF, mechanical/prosthesis heart valve, and DVT. In patients with prosthetic mechanical valves, the VKAs are the only choice for anticoagulation [65]. For AF patients, VKAs have been associated with a 64% reduced risk of stroke and 26% reduction in all-cause mortality, compared to control or placebo; however, VKAs use is also associated with a higher risk of ICH [66].

With the introduction of NOACs, which are associated with a significantly lower risk of ICH, the risk of anticoagulation-related ICH is perhaps decreasing compared to the VKA era [67]. The NOACs could be an optimal choice when considering anticoagulation resumption in patients with AF and incident ICH. Also, NOAC-related ICH seems to be less severe than that of warfarin, with smaller hematoma volumes, less hematoma expansion, and lower risk of poor functional outcome or death [68, 69].

The availability of reversal agents for the NOACs also is a consideration when using these drugs, given the possibility of rapid anticoagulation reversal in patients with incident ICH. However, the evidence of NOACs in patients with AF and recent ICH remains limited. Given the significantly lower risk of ICH in AF patients [61], the role of NOACs in these patients could be promising. In patients at high risk of DVT and PE, NOACs may also be an alternative to warfarin [70–75].

Heparin is also widely used as a temporary parenteral anticoagulant. One previous study demonstrated that low-dose heparin treatment after 48 h of onset in ICH patients was not associated with an increased hematoma growth and should be used for DVT and PE prophylaxis [76].

## Reducing the Risk of Recurrent ICH After Restarting OAC

After restarting OAC, the risk of ICH recurrence can be reduced through controlling those modifiable bleeding risk factors (Table 2) [77]. In patients in whom OAC resumption is not an option, left atrial appendage occlusion in patients with AF and the intra-venous filter in patients with DVT and PE are reasonable alternatives to reduce thromboembolic risk [78].

In patients treated with warfarin, well-controlled international normalized range (INR) was related to less major bleeding and thromboembolism compared with those without OAC (*p* < 0.01, respectively) [20]. Thus, in patients with mechanical prosthetic valves, for which only warfarin could be used, time in therapeutic range (ideally > 70%) is important. A lower target INR should not be encouraged, which is related to higher risk of thromboembolism in comparison to the guideline-recommended INR ranges [79].

## Conclusion

Given the observational nature of the studies regarding OAC resumption after ICH, there is uncertainty regarding resumption of OAC therapy and its timing. The limited high-quality evidence regarding this issue leads to limited evidence-based guidelines and inconsistency in clinical practice. There are randomized clinical trials ongoing, which may provide more information in the future [80]. For example, the APACHE-AF (Apixaban versus Antiplatelet drugs or no antithrombotic drugs after anticoagulation-associated intraCerebral HaEmorrhage in patient with Atrial Fibrillation) trial is a phase II, multicenter, randomized clinical trial, aiming to evaluate the risk of IS and recurrent ICH for patients restarted with anticoagulation [80]. This plans to include 100 AF patients with a history of recent ICH randomly assigned in a 1:1 ratio to apixaban or control [80]. The SoSTART (Start or Stop Anticoagulants Randomised Trial) trial is a multicenter, randomized, open, interventional trial, aiming to recruit 800 participants. This trial aims to test whether restarting full treatment dose OAC would result in a beneficial net reduction of all serious vascular events compared with not starting OAC [81].
